# Scrofulous Joint Inflammations

**Published:** 1882-02

**Authors:** 


### SCROFULOUS JOINT INFLAMMATIONS.

Prof. Hueter defines scrofulous joint inflammation as those in which the formation of masses of granulation tissue precedes suppuration, whilst in simple inflammation suppuration precedes the formation of granulation tissue.

He recommends the early injection by a Pravaz syringe of a three to five per cent. solution of carbolic acid into the incipient granulation tissue, whether it be situated in the synovial membrane or in the medulla of bone. If this does not prove satisfactory, he recommends early incision. It was pointed out by Mr. Morgan that in the hands of English surgeons complete rest and fixation of the scrofulous joint had been followed by a considerable amount of success; that the treatment of diseased synovial membrane by carbolic acid had not borne the fruit which had been expected of it; and that excision of joints, with the exception of the elbow had been repeatedly disappointing. Mr. Holmes also insisted that cases of joint disease, which everybody would agree to call scrofulous, had unquestionably recovered with rest, drainage, etc., and that the rule that if the injection of carbolic acid was not followed by improvement, excision should be adopted, was so absolute as to require much evidence to enforce its acceptance by English surgeons. Professor Hueter, in reply to the first observation, admitted that certain scrofulous inflammations of joints had subsided when at rest, but contended that they were cured by time, and not by rest. Dr. Sayre admitted that rest at the earliest time possible might effect a cure, but he objected to rest in bed in a hospital. He thought that only the inflamed joint should be at rest, that enough extension should be employed to take away pressure from the inflamed surface, and that as far as could be the investing tissues of capsule and the like should be exercised so as to keep up their nutrition.—*Trans. Int. Congress.*

